# Correlations between moral courage, moral sensitivity, and ethical decision-making by nurse interns: a cross-sectional study

**DOI:** 10.1186/s12912-023-01428-0

**Published:** 2023-08-08

**Authors:** Zongting Luo, Lan Tao, Carol Chunfeng Wang, Nan Zheng, Xiaolin Ma, Yi Quan, Jian Zhou, Zhen Zeng, Lijuan Chen, Yue Chang

**Affiliations:** 1 Department of Nursing, The Third People’s Hospital of Chengdu, Chengdu, China; 2 Australasian Academy of Cosmetic Dermal Science (AACDS), The University of Notre Dame Australia AU, Perth, Australia; 3 School of Nursing, Leshan Vocational and Technical College, Leshan, China; 4 Department of Rheumatology and immunology, The First Affiliated Hospital of Chengdu Medical College, Chengdu, China

**Keywords:** Fortitude, Moral awareness, Ethical principles, Ethical dilemmas, Nurse interns

## Abstract

**Background:**

Clinical decision-making involves ethical issues that become more and more complex. Nurse interns must be more skilled in making rational and timely decisions when facing ethical dilemmas. The contributing factors and their relationships that challenge ethical decision-making among nurse interns must be fully understood, as this level of knowledge can support the development of strategies and interventions that improve the ethical decision-making ability of nurse interns.

**Objective:**

This study examined the relationships between moral courage, moral sensitivity, and ethical decision-making by nurse interns. In addition, we investigated whether moral sensitivity mediates the relationship between moral courage and ethical decision-making.

**Design:**

A descriptive cross-sectional study using an online questionnaire.

**Setting:**

The study sampled nurse interns from Class iii Grade A general hospitals in Sichuan Province, China.

**Participants:**

A convenience sampling method was used to select 1334 nurse interns from March 2022 to May 2022.

**Methods:**

A general information questionnaire, the Nurses’ Moral Courage Scale (NMCS), the Chinese Moral Sensitivity Questionnaire (MSQ), and the Judgement About Nursing Decision (JAND) were used for data collection. Data was processed and analysed using SPSS 26.0 and Amos 28.0. Data analysis included descriptive statistics, Pearson correlation analysis, and structural equation modelling.

**Results:**

Moral courage was found to be positively correlated with ethical decision-making (*P* < 0.01). Moral sensitivity was also positively correlated with ethical decision-making (*P* < 0.01) and had a mediating effect on the relationship between moral courage and ethical decision-making (*P* < 0.01).

**Conclusions:**

The moral courage and moral sensitivity of nurse interns are positively correlated with ethical decision-making ability. Moral sensitivity significantly mediates the relationship between moral courage and ethical decision-making ability. The knowledge gained from this study can inform educational strategies and interventions in supporting the development of nurse interns’ ethical decision-making ability.

## Introduction

Due to the rapid development of medical technology and the increasing diversity of social and cultural values, the nursing profession’s ethical dilemmas have become increasingly complex and rational and sound decision-making is consequential to health outcomes [1, 2]. In 2021, the International Council of Nurses (ICN) issued the latest version of the Code of Ethics for Nurses. European and American countries such as the United Kingdom, Canada, and the United States have successively issued Codes of Ethics for their nurses and have continuously revised them to remain relevant in the fast-paced society [3]. Similarly, the Code of Ethics for nurses issued in China follows the basic principles and value of “patient-centred care” [3, 4]. The Code guides the nursing profession in China for its conduct, ethical decision-making, sustainable professional development, and, most importantly, better health outcomes.

Despite hardships and hazards in the face of moral challenges and dilemmas, ethical decision-making refers to not choosing unethical options and evaluating and selecting the best alternatives consistent with ethical principles [5]. Research has shown that ethical decision-making in nursing directly affects the quality of clinical care and the nurse-patient relationship [6]. Moral courage refers to the courage and ability of nurses to persist in acting according to their ethical values and principles despite the risk of foreseeable or actual negative consequences to themselves [7]. Escolar-Chua et al. studied 293 undergraduate nursing students in the Philippines and found that moral courage has a positive impact on ethical decision-making [8]. However, how moral courage influences ethical decision-making in nursing requires further exploration.

Moral sensitivity refers to sensitivity to ethical issues, including consideration of the ethical norms or principles [9]. Studies have shown that moral sensitivity is an important factor, for a strong moral sensitivity is conducive to more rational decision-making by nursing staff [10, 11]. Some scholars have reported preliminary evidence that moral courage positively affects the moral sensitivity of nurses [12].

Nurse interns are transitioning from campus to clinical work and act as a reserve force for the nursing team. Nurses raise concerns that nurse interns are often unable to make ethically rational decisions promptly when faced with ethical dilemmas due to a lack of soft nursing skills such as ethical awareness, observation and judgement [13, 14]. Therefore, it is imperative to improve the overall quality of the nursing team and healthcare services by developing ethical awareness and improving the ethical decision-making skills of nurse interns through improved training.

No literature reports on the relationships between moral courage, moral sensitivity, and ethical decision-making by nurse interns. Furthermore, the interaction between these factors needs to be clarified. Therefore, this study aimed to construct a structural equation model to explore these relationships in nurse interns and the mediating role of moral sensitivity in the relationship between moral courage and ethical decision-making.

## Methods

### Study design

A descriptive, cross-sectional study examined the relationship between nurse interns’ moral courage, moral sensitivity, and ethical decision-making. It also explored whether moral sensitivity mediated moral courage and ethical decision-making. All nurse interns consented and participated in this study voluntarily.

### Participants

This study was conducted from March 2022 to May 2022. By convenience sampling, nurse interns from 16 Class iii Grade A general hospitals in Sichuan Province were selected as the research participants. The inclusion criterion were the first-time internship in a hospital, and received the teaching of nursing ethics in the fourth semester of the school. The exclusion criteria was the termination of the internship for any reason.

### Data collection instruments

The data collection instruments used in this study included a general information questionnaire, the Nurses’ Moral Courage Scale (NMCS), the Chinese Moral Sensitivity Questionnaire (MSQ), and the Judgement About Nursing Decision (JAND).

The general information questionnaire was designed by the authors and collected the demographic characteristics of nurse interns, including their gender, age, education level, where they live, nationality, being the only child, experience as a student leader, willingness to choose the nursing major, attitude towards nursing profession, the way to obtain knowledge of nursing ethics, and whether they had received ethics-related training or continuing education during their internship.

The NMCS was compiled by Numminen et al. in 2018 and translated by Wang et al. in 2019 [15, 16]. This scale is used for the self-assessment of moral courage by nursing staff. It has 21 items and includes moral integrity, commitment to good patient care, compassion, and true presence with the patient, and moral responsibility. The NMCS uses the Likert 5-level scoring method, ranging from “completely non-conforming’’ to ‘‘completely conforming’’. These are assigned scores of 1 and 5, respectively, thus giving a total score range of 21–105. A higher score indicates a higher level of moral courage by the nursing staff. The Cronbach’s α of the total scale for NMCS was 0.905.

The MSQ was compiled by Lützén et al. in 2001 and was revised in 2006 [17]. Huang et al. published the Chinese translation and cultural adaptation of the MSQ in 2016 [18]. This scale is used to assess the moral sensitivity of nursing staff. The scale has nine items and consists of two dimensions: moral responsibility and strength and moral burden. The Likert 6-level scoring method is used, where 1 corresponds to “completely disagree” and 6 to “completely agree”, giving a total score range of 9*–*54. A higher score indicates a higher level of moral sensitivity by the nursing staff. The Cronbach’s α of the total scale for MSQ was 0.82.

Developed by Ketefian in 1981 and revised in 2007, the JAND is used to assess ethical decision-making by nursing staff [19]. Zhu et al. published the Chinese translation and cultural adaptation of the JAND in 2011 [20]. The scale includes two dimensions of ethical choice and action, comprising six ethical stories and two ethical scenarios. Each ethical issues involves several actions that need to be addressed based on “ethical choice” and “ethical action”. Ethical choice involves determining whether, under ideal circumstances, the nurse in the story should or should not perform each of the described actions. Ethical action involves determining when, given the constraints present on the unit or in the organisation, the nurse would realistically be likely to perform each of the described actions. The Likert 5-level scoring method is used, where 1 corresponds to “completely disagree” and 5 corresponds to “completely agree”. This gives a total score range of 76*–*380, with scores above 304 considered to represent a high level of ethical decision ability, scores between 228 and 304 as medium level, and scores of less than 228 as low level. The Cronbach’s α of the total scale for JAND was 0.876.

### Data collection

Data were collected through the Questionnaire Star website, a popular, powerful, and personalized questionnaire design system. Before the formal investigation, the authors contacted the nursing department staff at each participating hospital, who then acted as the investigators and received standardized training. During the data collection process, the nursing department staff explained to the nurse interns the purpose of the study and its implications. They used various recruitment methods, such as sending invitations via email, distributing the online survey platforms, providing clear participation instructions, and addressing any concerns participants may have. The nursing department staff communicated with participants throughout the data collection, providing updates and addressing concerns or questions. After receiving the link, the nurse interns completed the questionnaire anonymously using a computer, mobile phone and other network terminals. At the end of the survey, all questionnaires were exported from the Questionnaire Star website. A total of 1358 questionnaires were collected, and 24 were subsequently excluded due to obvious errors in information, unclear responses and uniform responses. This resulted in 1334 valid questionnaires and an effective recovery rate of 98.23%.

### Data analysis

The SPSS Statistics 26.0 software package was used for statistical analysis. The general data were described as frequency and composition ratio (%). Total scores for moral courage, moral sensitivity, and ethical decision-making, and the scores for each dimension, were described as the mean ± standard deviation. The Pearson coefficient analysed correlations between the three factors, with the significance level set at *P* < 0.05. SPSS Amos 28.0 was employed to develop a structural equation model to explore the relationships between moral sensitivity, courage, and ethical decision-making in nurse interns and the mediating role of moral sensitivity in these relationships. The bootstrap method was used to test the mediating hypothesis. A bias-corrected percentile bootstrap with a 95% confidence interval (CI) that did not contain 0 was considered to represent statistical significance.

## Results

### Participant characteristics

Among the 1334 eligible nurse interns in this study, 1213 (90.9%) were female, and 950 (71.2%) had received ethics-related training or continuing education during their internship. The demographic characteristics of the nurse interns who participated in this study are detailed in Table 1.

**Table 1:** The demographic characteristics of participants (N = 1334)

Variables	Category	N	%
gender	female	1213	90.93
age	16–18	87	6.52
	19–21	696	52.17
	22–24	551	41.31
education level	secondary vocational	78	5.85
	junior college	572	42.88
	undergraduate	684	51.27
area of residence	city	404	30.28
nationality	han nationality	1191	89.28
having siblings	yes	982	73.61
experience as a student leader	yes	965	72.34
reasons for choosing the nursing major	individual choice	337	25.26
	easy to obtain employment	436	32.68
	advice given by parents	455	34.11
	passive transfer to the nursing profession	106	7.95
attitude towards nursing profession	like	757	56.75
	doesn’t matter	438	32.83
	dislike	139	10.42
how many avenues exist for acquiring knowledge of nursing ethics	<2	389	29.16
	2–3	694	52.02
	≥ 4	251	18.82
received ethics-related training or continuing education during internship	yes	950	71.21

### Descriptive statistics of moral courage, moral sensitivity and ethical decision-making by nurse interns

The mean and standard deviation of the total score and of the dimension score for each study variable are shown in Table 2. The average scores were 75.88 (SD = 14.52) for moral courage, 39.72 (SD = 6.73) for moral sensitivity, and 272.08 (SD = 27.80) for ethical decision-making.

**Table 2:** Scores of moral courage, moral sensitivity, and ethical decision-making

Variables	Mean	SD	Min	Max
moral courage	75.88	14.52	21	105
moral integrity	24.95	5.09	7	35
commitment to good patient care	17.91	3.83	5	25
compassion and true presence with the patient	17.99	3.73	5	25
morality responsibility	15.03	3.02	4	20
moral sensitivity	39.72	6.73	9	54
moral responsibility and strength	22.46	4.02	5	30
moral burden	17.26	3.28	4	24
ethical decision-making	272.08	27.80	76	380
ethical choice	139.51	15.66	38	190
ethical action	132.57	14.83	38	190

### Correlation analysis of moral courage, moral sensitivity and ethical decision-making by nurse interns

Pearson correlation analysis revealed that the moral courage of nurse interns was positively correlated with both moral sensitivity (r = 0.613, *P* < 0.01) and ethical decision-making (r = 0.484, *P* < 0.01) (Table 3). Moreover, moral sensitivity was positively correlated with ethical decision-making (r = 0.515, *P* < 0.01).

**Table 3:** Correlations of moral courage, moral sensitivity, and ethical decision-making

Variables	1	2	3
1.moral courage	1.000		
2.moral sensitivity	0.613**	1.000	
3.ethical decision-making	0.484**	0.515**	1.000

** *P* < 0.01.

### Mediating role of moral sensitivity on moral courage and ethical decision-making

**Fig. 1:**
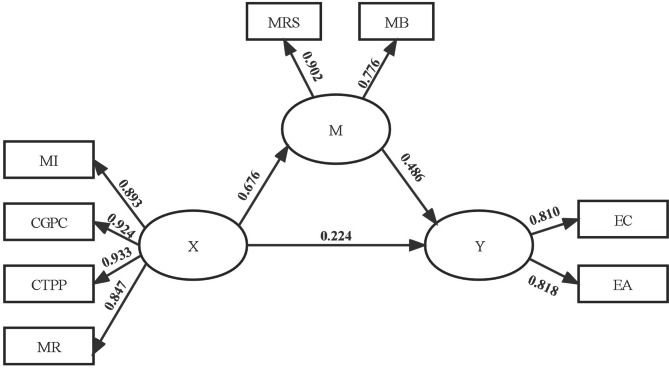
Standardized parameter estimates for the final structure model X: moral courage; MI: moral integrity; CGPC: commitment to good patient care; CTPP: compassion and true presence with the patient; MR: morality responsibility; M: moral sensitivity; MRS: moral responsibility and strength; MB: moral burden; Y: ethical decision-making; EC: ethical choice; EA: ethical action

The effect relationship amongst factors in the fitting model is shown in Fig. 1. Moral courage had a direct positive role on moral sensitivity (*β* = 0.676, *P* < 0.01), and moral sensitivity had a direct positive role on ethical decision-making (*β* = 0.486, *P* < 0.01). Furthermore, moral courage had a direct positive role on ethical decision-making (*β* = 0.224, *P* < 0.01).

To further examine the mediating role of moral sensitivity on moral courage and ethical decision-making, the bootstrap method was run 5000 times to obtain a value under 95% CI. As shown in Table 4, the indirect effect of moral courage on ethical decision-making was 0.329. The bootstrap 95% CI of 0.250*–*0.411 did not include a value of 0, thus demonstrating the statistical significance of the mediating role.

**Table 4:** The bootstrap analysis results of mediation effect test

	Standardized effect value	95% CI	
		Lower	Upper
indirect effect	0.329	0.250	0.411
direct effect	0.224	0.118	0.334
total effect	0.553	0.485	0.619

## Discussion

This study found positive correlations between moral courage, moral sensitivity, and ethical decision-making by nurse interns. Moreover, the results confirmed that moral sensitivity mediates between moral courage and ethical decision-making.

The present work also found that the moral courage of nurse interns was positively correlated with their ethical decision-making ability (*P* < 0.01). In other words, their higher level of moral courage was strongly correlated with their greater capable to make rational and better ethical decisions. The complexity of modern healthcare requires nurses to exercise moral courage to make rational and better ethical decisions [21]. Moral courage is an individual’s ability to overcome fear and adhere to their core values, directly or indirectly impacting ethical decision-making. Malik et al. highlighted the value of courage for rational decision-making in medical ethics [22]. In the clinical nursing environment, it is common for nurses to face moral dilemmas due to various pressures. A high level of moral courage can help them work consistently with ethical values, resist unethical behaviour, make rational nursing decisions, and reduce clinically-related risks. Studies [23–25] have reported that, in nursing practice, moral courage is needed due to many factors, such as the fear of being seen as a whistle blower, being punished for reporting medication errors by oneself or others, increasing technical or nursing costs, and lowering colleague expectations, which sometimes leave ethics to the side. Thus there are many reasons to rock the boat of moral courage. If the boat of moral courage is rocked, it is not conducive to making rational nursing decisions, which will have a negative impact on the quality and safety of care. Some studies have shown that psychological empowerment, a good working environment and strong positive organizational culture can promote moral courage in nurses [26, 27]. It was suggested that nurse interns learn to self-regulate, overcome destructive emotions, and increase risk tolerance. Nursing managers should therefore strive to improve the organization’s ethical culture, reduce moral distress, and create a supportive environment that nurtures and rewards honest and courageous behaviour. In summary, improving the moral courage of nurse interns can help them make more rational ethical decisions.

The results of this study showed that the moral sensitivity of nurse interns was positively correlated with their ethical decision-making ability (*P* < 0.01). This is similar to a previous study [28]. Moral sensitivity is defined as the ability to keenly perceive and explain moral problems or situations [29]. The occurrence of ethical issues in clinical nursing practice is rising due to the increasing complexity of the medical environment. Nurses must have high moral sensitivity to help them better understand and deal with clinical ethical issues and to make morally appropriate nursing decisions. As early as 2005, it was reported that nurses with higher levels of moral sensitivity are better able to perceive the needs of patients, thereby providing a better quality of nursing care [30]. In a follow-up study of 946 undergraduate nursing students in South Korea, Park et al. found that moral sensitivity was related to moral reasoning, thus affecting their ethical decision-making ability [31]. This further confirms that moral sensitivity is closely related to ethical decision-making ability. Moral education and the moral environment are two important factors that influence moral sensitivity. It has been suggested that nurse interns’ moral education should be strengthened to help them understand and analyse clinical ethical issues [23, 31, 32]. Creating a positive moral environment and enhancing the moral sensitivity of nurse interns should become a priority of nursing education reform in schools or hospitals.

This study found that moral sensitivity was a significant mediator of the relationship between moral courage and ethical decision-making of nurse interns. The indirect effect that moral sensitivity had on ethical decision-making via moral courage accounted for 59.5% of the total impact observed in the present study. This indicates that moral sensitivity is important in helping nurse interns make rational ethical decisions. A possible explanation is that individuals with moral courage are not afraid to be firm in the face of moral dilemmas [33]. Nurse interns endowed with moral courage can identify ethical issues, analyse the existing ethical situation, increase their moral sensitivity, and take rational moral decisions and actions in a complex nursing care environment. Moral sensitivity is a prerequisite for ethical decision-making. If moral courage is lacking and ethical dilemma in the situation cannot be sensitively perceived, then ethical decision-making cannot be made, let alone ethical actions [34]. To enhance the ethical decision-making ability of nurse interns, we propose that universities and clinical institutions in China should not focus solely on direct training in this ability. Instead, they should also promote the cultivation of moral courage and sensitivity through moral education, ethical dilemma simulation, and the construction of ethical environments. For example, lectures on ethical knowledge; patient activity, through playing the role of patients, empathizing with patients and learning to take the patient’s perspective; creating an honest atmosphere, with key figures such as mentors playing the role of good role models, and commending and encouraging nurses who perform well in nursing ethics. Improving the moral courage and sensitivity of nurse interns and helping them make rational ethical decisions, should gradually lead to a relatively complete, scientific and systematic training system for ethical decision-making.

## Limitations

Due to time and human resource limitations, the research subjects for this study were all from Sichuan province level Class iii Grade A comprehensive hospitals. The scope of the study may be seen as limited. However, the sample of hospitals included represents the total “population” of the same level (class iii Grade) hospitals in China. The research subjects are not different from other provinces in China and are similar to hospitals in most provinces. The type of hospitals chosen is also similar to other hospitals in China. Another limitation was that a longitudinal study to examine the timeline of changes in nursing ethical decision-making ability was not performed. Therefore, future studies should conduct longitudinal studies. Also, an in-depth study on the causal relationship between relevant relationship variables will also be the direction of future research.

## Conclusions

The results of this study showed that the nurse interns’ moral courage and moral sensitivity are positively correlated with ethical decision-making ability. Moral sensitivity significantly mediates the relationship between moral courage and ethical decision-making. Educating and training moral courage and sensitivity may lead to better decision-making, but it needs to be explored more.

## Data Availability

The datasets used and/or analyzed during the current study are available from the corresponding author on reasonable request.
